# Oct4 upregulates osteopontin via Egr1 and is associated with poor outcome in human lung cancer

**DOI:** 10.1186/s12885-019-6014-5

**Published:** 2019-08-09

**Authors:** Yin-Hsun Feng, Yu-Chu Su, Shuo-Fu Lin, Pey-Ru Lin, Chao-Liang Wu, Chao-Ling Tung, Chien-Feng Li, Gia-Shing Shieh, Ai-Li Shiau

**Affiliations:** 1 Division of Hematology and Oncology, Department of Internal Medicine, Chi-Mei Medical Center, 901 Chung-Hwa Road, Tainan, 71004 Taiwan; 2 Department of Nursing, Chung Hwa University of Medical Technology, Tainan, Taiwan; 3 Department of Biochemistry and Molecular Biology, College of Medicine, National Cheng Kung University, Tainan, Taiwan; 4 Department of Otolaryngology, National Cheng Kung University Hospital, College of Medicine, National Cheng Kung University, Tainan, Taiwan; 5 Department of Microbiology and Immunology, College of Medicine, National Cheng Kung University, 1 University Road, Tainan, 70101 Taiwan; 6 Department of Pathology, Chi-Mei Medical Center, Tainan, Taiwan; 7 Department of Urology, Tainan Hospital, Ministry of Health and Welfare, Executive Yuan, Tainan, Taiwan

**Keywords:** Oct4, Egr1, Osteopontin, Lung cancer, Metastasis

## Abstract

**Background:**

Roles of cancer stem cells and early growth response gene 1 (Egr1) in carcinogenesis have been extensively studied in lung cancer. However, the role of Egr1 in the metastasis of lung cancer remains undetermined, especially in regard to stem cell-related pathways.

**Methods:**

Egr1, osteopontin (OPN) and Oct4 expression in human lung cancer was determined by performing immunohistochemistry. Immunoblotting, ELISA, luciferase reporter assay, chromatin immunoprecipitation assay and RT-PCR were performed to validate the regulation of Oct4-Egr1-OPN axis. Moreover, the effect of Oct4-Egr1-OPN axis on lung cancer progression was evaluated by cell migration assay and mice study.

**Results:**

We detected Oct4, Egr1, and OPN expression in clinical specimens from 79 lung cancer patients, including 72 adenocarcinomas and 7 squamous cell carcinomas. High expression of Oct4, Egr1, and OPN accounted for 53, 51, and 57% of the patients, respectively. All of the three biomarkers were positively correlated in clinical human lung cancer. Patients with high expression of OPN were significantly associated with shorter disease-free survivals than those with low expression of OPN (*p* < 0.05). In lung cancer cells, Oct4 transactivated the *Egr1* promoter and upregulated Egr1 expression. In a human lung cancer xenograft model, Oct4-overexpressing tumors expressed elevated levels of Egr1. Furthermore, overexpression of Oct4 in lung cancer cells increased the metastatic potential.

**Conclusions:**

Egr1 exerts a promoting effect on cancer metastasis in Oct4-overexpressing lung cancer. Thus, therapeutic strategies targeting the Oct4/Egr1/OPN axis may be further explored for the treatment of lung cancer, especially when lung cancer is refractory to conventional treatment due to cancer stem cells.

**Electronic supplementary material:**

The online version of this article (10.1186/s12885-019-6014-5) contains supplementary material, which is available to authorized users.

## Background

Lung cancer is regarded as a leading cause of human cancer deaths, responsible for nearly 1.59 million deaths worldwide. More than half of patients diagnosed with lung cancer die within one year after diagnosis [1, 2]. The poor outcome of lung cancer may be attributable to a high incidence of metastasis at diagnosis and a high frequency of recurrence after curative resection. Stem cells regenerate all tissues and evoke self-renewal in order to produce an adequate supply of cells for tissue repair. Furthermore, emerging evidence supports the cancer stem cell hypothesis that malignant tumors are initiated and maintained by the progenitor cells within tumors that share similar biological characteristics to normal adult stem cells [3]. Several biomarkers have been used in stem cell research to identify the characteristics of lung cancer stem cells. Among these, CD44 and CD133 are most commonly used [4]. CD44 is a transmembrane glycoprotein that binds hyaluronic acid, which is an abundant polysaccharide in stem cells and in cells with coexpression of CD44 and CD90. This then leads to an increase in the mRNA levels of the embryonic stem cell-related genes Nanog and Oct4 [5, 6]. We have previously shown that treatment with suboptimal doses of cisplatin can induce drug resistance and increase the expression of CD44, CD133, ABCG2, and osteopontin (OPN) in human lung cancer cells [7]. Oct4, a stem cell marker [8, 9], is expressed in several human cancers, but not in normal somatic tissues [10–12]. In clinical samples, we have previously reported that bladder tumors with intense expression of Oct4 are associated with further disease progression, greater metastasis, and shorter cancer-related survival compared to those with moderate and low expression of Oct4 [12]. Furthermore, we have shown that treatment with cisplatin increases CD44-positive bladder cancer cells expressing Oct4, representing cancer stem-like cell subpopulation, which contributes to drug resistance and tumor recurrence [13]. In lung cancer-derived CD133-positive cells, Oct4 expression maintains the self-renewable, cancer stem-like, and chemoradio-resistant properties of CD133-positive cells [14]. We have reported that oncolytic adenoviruses driven by the *Oct4* promoter or Oct4 response element (ORE) exert antitumor activities against blander cancer that expresses Oct4, validating Oct4 as a potential therapeutic target for cancer [12, 15].

Early growth response gene 1 (Egr1), a C2H2-zinc finger-containing transcriptional factor, is a member of the immediate-early gene family that is promptly activated by various mitogens, growth factors, apoptotic signals, and ischemia in different tissues [16, 17]. Furthermore, Egr1 is involved in the regulation of cell growth, differentiation, and apoptosis [18, 19]. In prostate cancer, Egr1 inhibits IL-8-mediated invasion of prostate cancer cells through suppressing the Egr1/NF-κB pathway [20]. Moreover, Egr1 selectively increases the activation of activation protein-1 (AP-1) and NF-κB, leading to the induction of proliferation and anchorage independence in prostate cancer cells [21]. The role of Egr1 in lung cancer has been elucidated. The wild-type epidermal growth factor receptor or MET receptors can sustain the survival of lung cancer cells via Egr1 and/or the extracellular signal-regulated protein kinase (ERK) 1/2. Akt activation and Egr1 overexpression prevent cell death induced by combined anaplastic lymphoma kinase/receptor tyrosine kinase inhibition [22]. Using knockdown and microarray analyses of mouse embryonic stem cells, Loh et al. identified 4711 differentially expressed genes in Oct4 knockdown cells [23]. Among these genes, the *Egr1* gene was downregulated. Nevertheless, the interaction and clinical significance of Oct4 and Egr1 remain unclear in cancer cells.

OPN is a multifunctional cytokine that contributes to cell proliferation, survival, drug resistance, invasion, and stem cell behavior [24]. It has been documented that Egr1 upregulates OPN through direct binding to its promoter, and OPN upregulates Egr1 via the ERK pathway [25]. OPN has been linked to sustaining hematopoietic stem cell polarity and attenuating the aging of old hematopoietic stem cells [26]. However, the interaction between Egr1 and OPN in lung cancer remains largely unclear. In the present study, we aimed to determine the association and clinical relevance of Oct4, Egr1, and OPN in lung cancer and to identify the related pathways and potential therapeutic strategies for the treatment of lung cancer.

## Methods

### The study population and tumor samples

A total of 79 patients were included in this study. The patients were diagnosed with lung cancer between September 1998 and March 2010 at the Chi-Mei Medical Center, Department of Pathology, Tainan, Taiwan. There were no restrictions on gender, histological subtype, or stage. The patients were surgically treated and had primary lung cancers, including 72 adenocarcinomas and 7 squamous cell carcinomas.

### Cell lines and plasmids

Human lung cancer H1299 and A549 cell lines were purchased from the Bioresource Collection and Research Center (Food Industry Research and Development Institute, Hsinchu, Taiwan). These cell lines have been authenticated by the provider and tested for the contamination of mycoplasma. Cells were cultured at 37 °C in an atmosphere of 5% CO_2_ in DMEM (Hyclone, Logan, UT, USA) supplemented with 10% cosmic calf serum (CCS) (Hyclone). The Oct4 cDNA was cloned into *Bam*HI/*Sal*I sites of pCMV-tag2B (Stratagene, La Jolla, CA, USA) to generate a Flag-tagged Oct4 expression plasmid (pCMV-tag2B-Oct4). The *Egr1* promoter construct pGL2-Egr1p encompassing Egr1 promoter (− 710 ~ + 9 relative to the translational start site at + 1) was modified to generate two deletion mutants [27]. The 220-bp *Egr1* promoter region (from − 116 to − 336) was excised from pGL2-Egr1p by digestion with *Eag*I, filled-in the cohesive ends with Klenow fragment of DNA polymerase, and self-ligated with T4 DNA ligase to generate pGL2-Egr1p (− 116 ~ − 336). Similarly, the 469-bp Egr1 promoter region (from − 183 to − 652) was excised from pGL2-Egr1p by digestion with *Apa*I, rendering blunt-ended with T4 DNA polymerase, and self-ligated to generate pGL2-Egr1p (− 183 ~ − 652). The lentiviral vector pSin-EF2-Oct4-Pur encoding Oct4 was purchased from Addgene (Cambridge, MA, USA), and the control vector pSin-EF2-Pur encoding no transgenes was described previously^18^. For the knockdown experiments, the pLKO.1-puro-based lentiviral vectors expressing short hairpin RNA (shRNA), specific for Egr1 (TRCN000013833~7) and luciferase (Luc) (TRCN0000072246), were obtained from the National RNAi Core Facility, Academia Sinica, Taiwan. Various recombinant lentiviruses, including LV.Oct4, LV.Null, LV.shEgr1, and LV.shLuc, were produced as previously described [28].

### Immunohistochemistry

Immunohistochemistry of paraffin sections was carried out using the Novolink Polymer Detection System (Novocastra, Newcastle, UK) according to the manufacturer’s instructions. Paraffin-embedded sections were first deparaffinized and then hydrated. After microwave-stimulated antigen retrieval, as required, slides were washed for 5 min in phosphate-buffered saline (PBS). Endogenous peroxidase was neutralized using a peroxidase block for 5 min. Following incubation, the sections were washed in PBS (2 × 5 min washes). The sections were incubated with a protein block for 5 min and then washed again in PBS (2 × 5 min washes). The primary rabbit polyclonal antibodies used were anti-human-Oct4 (GTX101497, GeneTex, San Antonio, TX, USA), anti-Egr1 (ab54966, Abcam, Cambridge, UK), and anti-OPN (ab8448, Abcam). After serial incubation with primary antibodies overnight at 4 °C, the sections were washed in PBS (2 × 5 min washes) and incubated with Novolink Post Primary for 10 min. After incubation, the slides were washed in PBS (2 × 5 min washes), incubated with Novolink Polymer for 10 min, and then washed in PBS (2 × 5 min with gentle rocking on an orbital shaker). A 3–3′- diaminobenzidine working solution was applied to the slides for 3 min. The slides were washed in PBS, counter-stained with hematoxylin for 1 min, and then washed in distilled water for 5 min before dehydration, clearing, and mounting. Microscopic fields with the highest degree of immunoreactivity were selected for analysis. The score of intensity represented the mean-staining intensity of the positive tumor cells. It was classified as follows: negative, 0; weak, 1; moderate, 2; and strong, 3. The expressions of indicated proteins were categorized as “low” for negative or weak staining versus “high” for moderate or strong staining.

### ELISA and luciferase reporter assay

The expression level of OPN was determined using an ELISA (DY1433, R&D, Minneapolis, USA) according to the manufacturer’s instructions. Briefly, each well of 96-well ELISA plates (Maxisorp, Nunc, Denmark) was coated with the monoclonal capture antibody (360 μg/ml), and were subsequently incubated at 4 °C overnight. After 2 h, the plates were blocked at room temperature with BSA (Sigma-Aldrich, St. Louis, MO, USA), followed by incubation with samples at room temperature for 2 h. After addition of detection antibodies for 2 h, streptavidin-HRP was added to the plates, and 3,3′,5,5′- tetramethylbenzidine was used as the HRP substrate. The reaction was terminated using 1 M H_2_SO_4_. The optical density (OD) value was detected with a microplate spectrophotometer at 450 nm.

To analyze the *Egr1* promoter activity, H1299 cells (5 × 10^4^) grown in 24-well plates were cotransfected with 1 μg of pGL2-Egr1p, a firefly luciferase reporter plasmid driven by the *Egr1* promoter, and 0.5 μg of pTCY-Renilla, a *Renilla* luciferase reporter plasmid driven by the rat β-actin promoter, using polyethylenimine (PEI). After 24 h, the cells were transduced with LV.Oct4 or the control vector LV.Null. In addition, A549 stable clones overexpressing Oct4 (A549-Oct4) and vector control clones were established using LV.Oct4 and LV.Null transduction. The stable clones were then cotransfected with pGL2-Egr1p and pTCY-Renilla. Lysates were harvested from H1299 and A549 cells after 48 h, and their firefly and *Renilla* luciferase activities were determined with a dual-luciferase reporter assay (Promega, Madison, WI, USA). The firefly luciferase activity was normalized to *Renilla* luciferase activity.

### Chromatin immunoprecipitation (ChIP) assay

A ChIP assay was performed using the EZ-CHIP kit (Millipore, Bedford, MA, USA) as previously described [28]. Briefly, the cells were fixed using formaldehyde (1%), followed by sonication, alternating 30 s pulses 10 times in order to shear DNA to lengths between 500 and 1,000 bp. Then, anti-Oct4 antibody (Santa Cruz Biotechnology, Santa Cruz, CA, USA) was used to immunoprecipitate Oct4-bound DNA. The DNA was extracted and used for PCR. The primer sequences were 5′- AGGATCCCCCGCCGGAACAA-3′ (forward) and 5′-GAGTTCCCGCGTTGCCCCTC-3′ (reverse).

### Immunoblot analysis

Immunoblotting was performed using a standard method. The primary antibodies used for immunoblotting included Egr1 (C-19, sc-189, 1:1000, Santa Cruz), Oct-3/4 (C-20, sc-8629, 1:100, Santa Cruz), and β-actin (Sigma-Aldrich). After incubation with the secondary antibodies for 2 h, protein-antibody complexes were detected using the ECL system (Millipore) and then visualized with a Biospectrum AC imaging system (UVP, Cambridge, UK).

### RT-PCR analysis

Total RNA was isolated using Trizol (Gibco BRL, Grand Island, NY, USA), and 2 μg of RNA was used for the cDNA synthesis with a Reverse-iT TM 1st Stand Synthesis RT-PCR kit (AB gene, Epsom, UK) according to the manufacturer’s instructions. The following primers were used for RT-PCR: Egr1, 5′-CGGCAGAAGGACAAGAAAGCAGAC-3′ (forward) and 5′- GGGGAAGTGGGCAGAAAGGATTG-3′ (reverse); Oct4, 5′- CTCCGAGTGTGGTTCTGTA-3′ (forward) and 5′-CTCAGTTTGAATGCATGGGA-3′ (reverse); GAPDH, 5′-GCCATCACTGCCACCCAG-3′ (forward), and 5′- TCTTACTCCTTGGAGGCCATGT-3′ (reverse).

### Boyden chamber assay

Cell migration was assessed using a modified Boyden chamber assay with 8-μm pore poly-carbonate filters coated with gelatin as previously described [29]. A549-Oct4 and vector control cells (2 × 10^5^ cells/well) were individually added to the upper chamber containing serum-free DMEM. The lower chamber containing DMEM supplemented with 10% CCS. After incubation for 8 to 10 h, the filter was then fixed with 100% methanol and stained with a Giemsa solution. Cells on the upper surface of the filter were scraped with cotton buds. Migratory cells on the underside of the filter were then photographed and counted under a microscope.

### Animal studies

Eight-week-old male NOD/SCID mice were subcutaneously inoculated with 5 × 10^6^ of A549-vector or A549-Oct4 cells at day 0. Four mice were used for each group. The mice were sacrificed at day 60. Lung tissue sections were stained with H&E for analyzing the numbers of nodules. The euthanasia of these mice was conducted by cervical dislocation. All experimental procedures were approved by the Laboratory Animal Care and Use committee of National Cheng Kung University and performed in accordance with the approved guidelines.

### Statistical analysis

The categorical distribution difference for Egr1 or OPN between low and high Oct4 or Egr1, respectively, was calculated using Fisher’s exact test. The Kaplan-Meier survival curves with log-rank test were plotted to perform the survival trend difference. After checking with Shapiro-Wilk test or the Kolmogorov-Smimov test, all interesting variables did not present as normality (all *p* < 0.05). The Kruskal-Wallis test was used to estimate the difference among three groups with post-hoc analysis using the Bonferroni error correction (Fig. 2a, left panel). In addition, the Wilcoxon rank-sum test was used to the difference between two groups. The GraphPad PRISM 6.0 (GraphPad Software, San Diego, CA, USA) and SigmaPlot 12.0 software (Systat Software. Inc., San Jose, CA, USA) were applied to the statistical plots. All statistical analyses were performed with STATA (version 12; StatCorp, College Station, TX, USA). The statistically significant was set as *p*-value < 0.05.

## Results

### Expression of Oct4 is positively correlated with Egr1 and OPN expression in human lung cancer

**Fig. 1:**
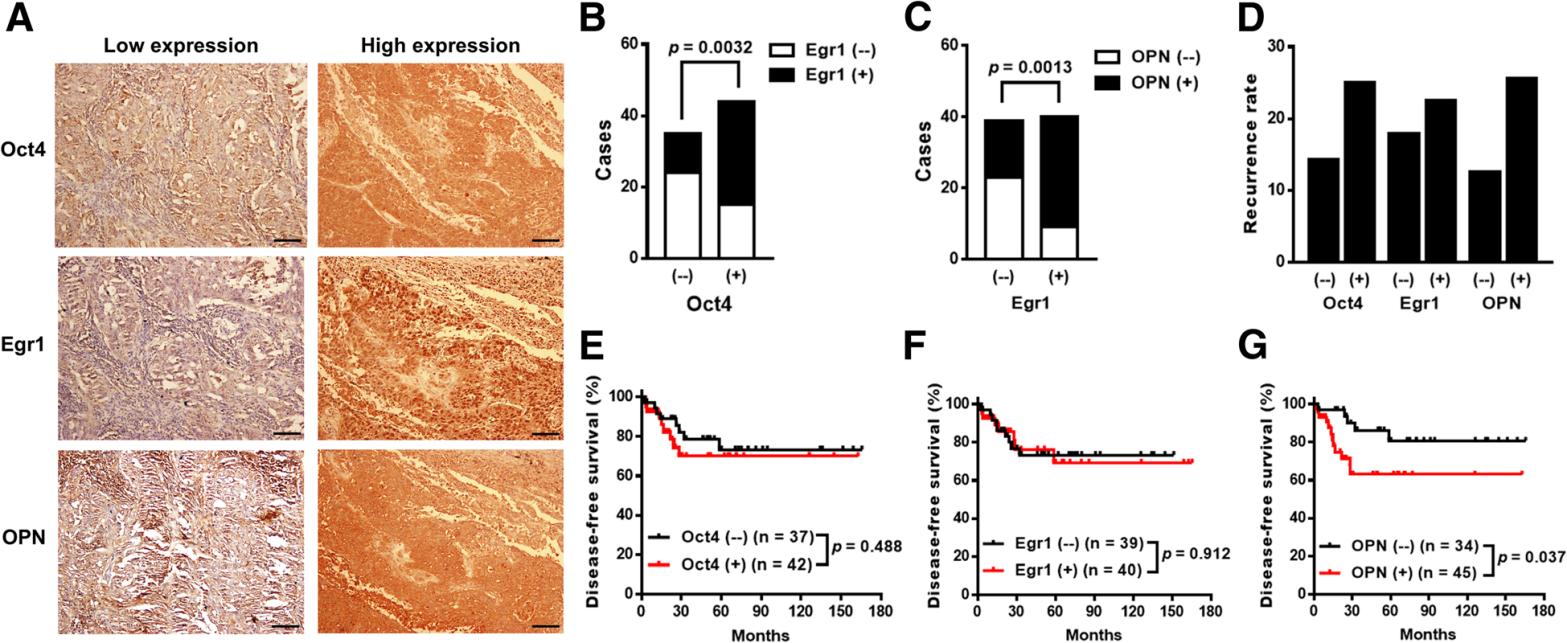
Oct4, Egr1, and OPN are overexpressed in lung cancer and Egr1 expression is correlated with shorter survival. **a**, Representative staining for Oct4, Egr1, and OPN in human lung tumors graded as low and high expression. **b**, Numbers of tumors expressing high or low levels of Egr1 in tumors with high or low Oct4 expression. **c**. Numbers of tumors expressing high or low levels of OPN in tumors with high or low Egr1 expression. **d**, Tumor recurrence rates between high- and low- expression of Oct4, Egr1, and OPN in lung cancer patients, none of them reached statistical significance (Oct4: *p* = 0.273; Egr1: 0.261; OPN: 0.253). **e**-**g**, Kaplan-Meier curves for disease-free survivals in patients with high (red line) and low (black line) Oct4 (**e**), Egr1 (**f**), and OPN (**g**) expression

Seventy-nine patients with stage Ib to stage IIIA lung cancer were included. Their tumor samples were immunohistochemically examined for the expression of Oct4, Egr1, and OPN. Complete surgical resection was the sole treatment modality for these patients. The demographic information of these patients is shown in Additional file 1: Table S1. The immunohistochemical staining for each specific protein was categorized into either high or low expression, according to the immunoreactive intensity (Fig. 1a). High expression of Oct4, Egr1, and OPN accounted for 42 (53%), 40 (51%), and 45 (57%) of the 79 patients, respectively. More Oct4 high-expressing tumors belonged to Egr1 high-expressing tumors, whereas tumors with low Oct4 expression consisted of more Egr1 low-expressing tumors (*p* < 0.005) (Fig. 1b). Similarly, most of the Egr1 high-expressing tumors, but not the Egr1 low-expressing tumors, expressed high levels of OPN (*p* = 0.001) (Fig. 1c). Notably, tumors with higher expression of Oct4, Egr1, and OPN were not associated with higher recurrence rates compared with those with lower expression of Oct4, Egr1, and OPN, respectively (Fig. 1d). In the disease-free survival analysis, there were no significant differences between Oct4 (Fig. 1e) or Egr1 (Fig. 1f) low-expressing and high-expressing tumors. However, Kaplan-Meier analysis shows that patients with OPN high-expressing tumors had significantly lower disease-free survival rates than those with OPN low-expressing tumors (*p* = 0.037) (Fig. 1g). Furthermore, we assessed the correlations among Oct4, Egr1, and OPN with the disease-free survival using a univariate survival analysis. Collectively, these results suggest the oncogenic roles for these three proteins and their potential interactions in human lung cancer.

### Oct4 transactivates the *Egr1* promoter encompassing the ORE in lung cancer cells

**Fig. 2:**
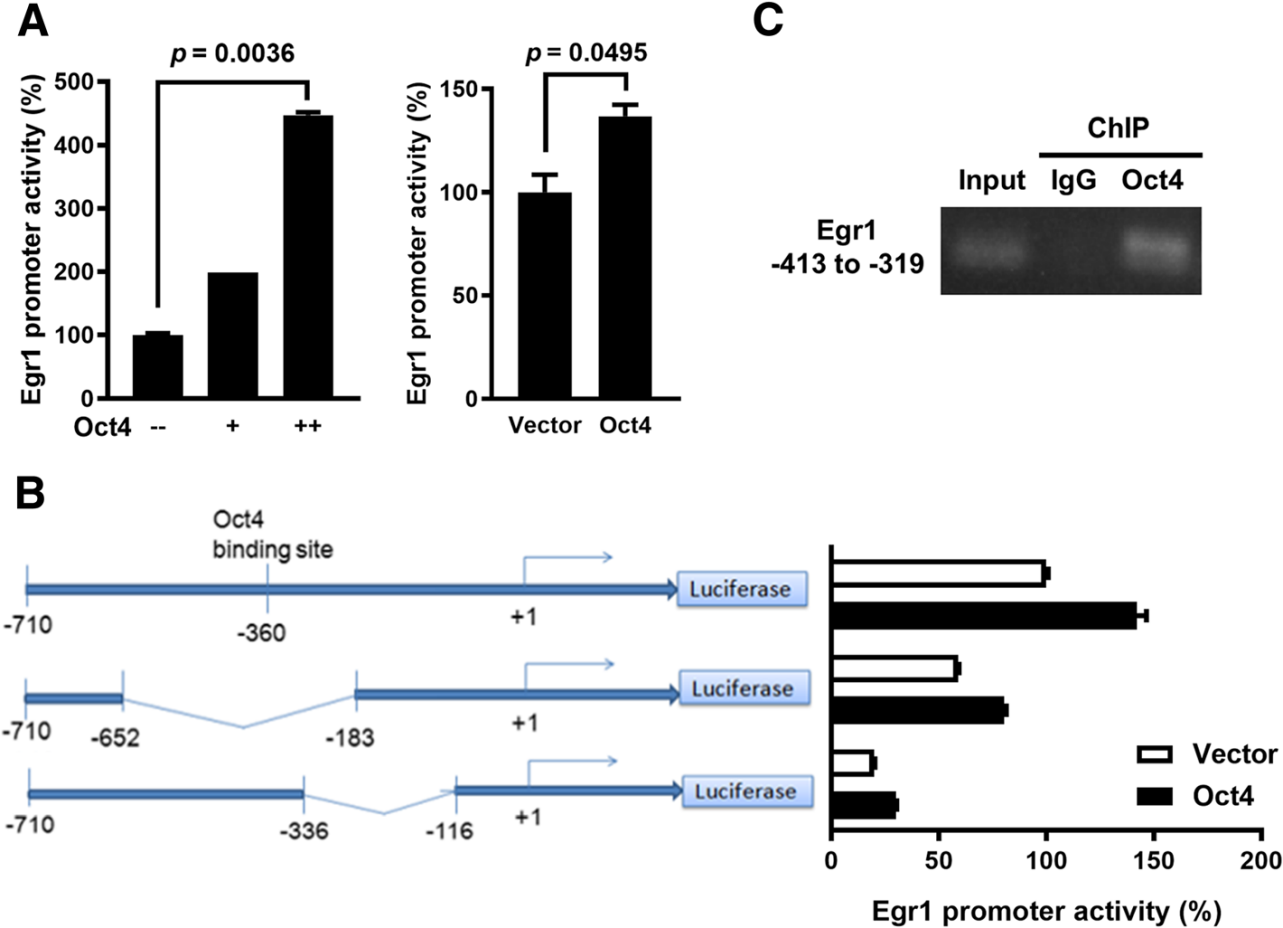
Oct4 binds to the *Egr1* promoter to transactivate Egr1 in lung cancer cells. **a**, H1299 cells that had been cotransfected with pGL2-Egr1p and pTCY-Renilla for 24 h were transduced with LV.Oct4 or LV.Null (left panel). A549 cells stably overexpressing Oct4 and vector control cells were cotransfected with pGL2-Egr1p and pTCY-Renilla (right panel). After 48 h, cell lysates were harvested, and their firefly and *Renilla* luciferase activities were determined. The ratio of firefly luciferase activity to *Renilla* luciferase activity of vector control cells was set to 100, and the values are presented as relative promoter activities. **b**, Schematic representation of two deletion constructions containing different lengths of the *Egr1* promoter (left panel). Numbering is relative to the translational start site at + 1. H1299 cells were cotransfected with full-length or different deletion constructs of the *Egr1* promoter and pTCY-Renilla. After 24 h, the cells were transfected with pCMV-tag2B-Oct4 or the control vector pCMV-tag2B. Cell lysates were assessed for firefly and *Renilla* luciferase activities 48 h later. RLU, relative luciferase unit. **c**, ChIP analysis showing direct binding of Oct4 to the Oct4 response element (ORE)-containing region in the *Egr1* promoter. Cross-linked chromatin of A549 cells was immunoprecipitated with anti-Oct4 or anti-IgG antibody combined with protein G agarose beads, followed by PCR amplification of the *Egr1* promoter region encompassing the ORE (between − 309 to − 413 bp) region. Data are mean ± SEM. *, *p* < 0.05; ***, *p* < 0.001

Oct4 regulates downstream genes through its binding to the Oct4 response element in ES and cancer cells [30]. To examine whether Oct4 upregulated Egr1 expression by transactivating the *Egr1* promoter, we used a luciferase reporter assay to detect the *Egr1* promoter activity in lung cancer cells after lentivirus-mediated overexpression of Oct4. The *Egr1* promoter activity was increased after transduction with different doses of LV.Oct4, but was unaffected by transduction with the vector control LV.Null in H1299 cells (Fig. 2a, left). Such an effect was also found in A549 cells transduced with LV.Oct4 (Fig. 2a, right). A subsequent analysis with the bioinformatics software Vector NTI (Invitrogen, Carlsbad, USA) revealed that the *Egr1* promoter sequence at − 360 bp has a 75% similarity with the Oct4 binding sequence “ATGCAAAT” [31]. We deleted the promoter regions from − 652 to − 183 bp and from − 336 to − 116 bp, and compared the transcriptional activities of deletion mutant and full-length promoters. Deletion of the promoter region between − 652 and − 183 bp dramatically abolished the responsiveness of the *Egr1* promoter to Oct4 in H1299 cells, whereas deletion between − 336 and − 116 bp still retained, albeit reduced, its responsiveness to Oct4 (Fig. 2b). Furthermore, a ChIP assay confirmed the binding of Oct4 to the DNA fragment encompassing the region from − 413 to − 309 bp within the *Egr1* promoter (Fig. 2c). Taken together, these results indicate that Oct4 directly binds to the *Egr1* promoter and transactivates the Egr1 gene expression.

### Overexpression of Oct4 increases the mRNA and protein levels of Egr1 in lung cancer cells

**Fig. 3:**
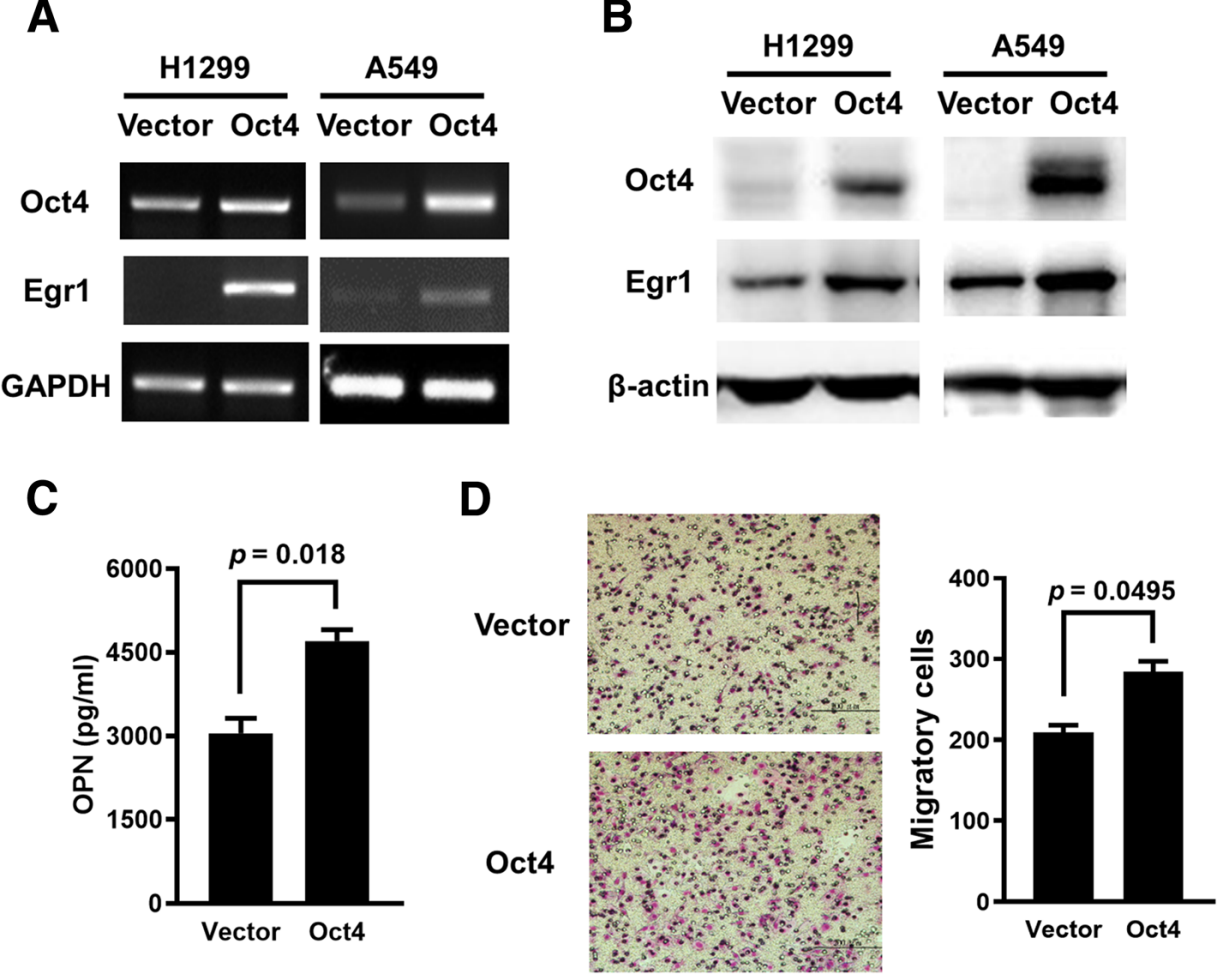
Overexpression of Oct4 enhances Egr1 expression, OPN production, and migratory capability of human lung cancer cells. **a** and **b**, H1299 cells (left panel) were transiently transfected with 6 μg of pSin-EF2-Oct4-Pur or the control plasmid pSin-EF2-Pur, and their RNA (**a**) and protein (**b**) levels of Oct4 and Egr1 were analyzed at 48 post-transfection by RT-PCR (**a**) and immunoblot (**b**) analysis. A549 cells (right panel) stably overexpressing Oct4 and vector control cells were examined for Oct4 and Egr1 expression by RT-PCR (**a**) and immunoblot (**b**) analysis. Expression of GAPDH (**a**) and β-actin (**b**) served as the loading control. **c**, The levels of OPN in the supernatants of Oct4-overexpressing and control A549 cells were quantified by ELISA. **d**, Migratory capabilities of A549-Oct4 and A549-vector cells were detected by a Boyden chamber assay. Cells that migrated through the membrane of the lower surface in the Boyden chamber were stained with Giemsa solution and visualized by light microscopy and photographed (left panel), and three fields in each membrane were counted (right panel). Data are mean ± SEM. **, *p* < 0.01

Previous microarray data have revealed that knockdown of Oct4 in mouse embryonic stem cells downregulates the Egr1 mRNA level [23]. To explore the effect of Oct4 on the Egr1 pathway, lung cancer cells were transfected with pSin-EF2-Oct4-Pur or the control plasmid pSin-EF2-Pur, and the expression of Egr1 was detected. Overexpression of Oct4 upregulated the expression of Egr1 at the mRNA (Fig. 3a) and protein (Fig. 3b) levels in H1299 and A549 cells. Egr1 has been shown to bind to the *OPN* promoter and upregulate OPN expression in rat vascular smooth muscle cells [25]. Moreover, overexpression of OPN enhanced the migratory capability of vascular smooth muscle cells, which could be reduced by pharmacological inhibition of the OPN-Egr1 pathway [32]. We further investigated whether Oct4 upregulated OPN expression through Egr1. Figure 3c shows that A549-Oct4 cells secreted more OPN than their control counterparts, as analyzed by ELISA (Fig. 3c). A Boyden chamber assay revealed that overexpression of Oct4 enhanced the migratory capability of A549 cells (Fig. 3d). Taken together, the expression level of Oct4 affects the expression of Egr1 and its downstream effecter OPN.

### Overexpression 006Ff Oct4 promotes metastasis in vivo and increases migration of lung cancer cells through Egr1

**Fig. 4:**
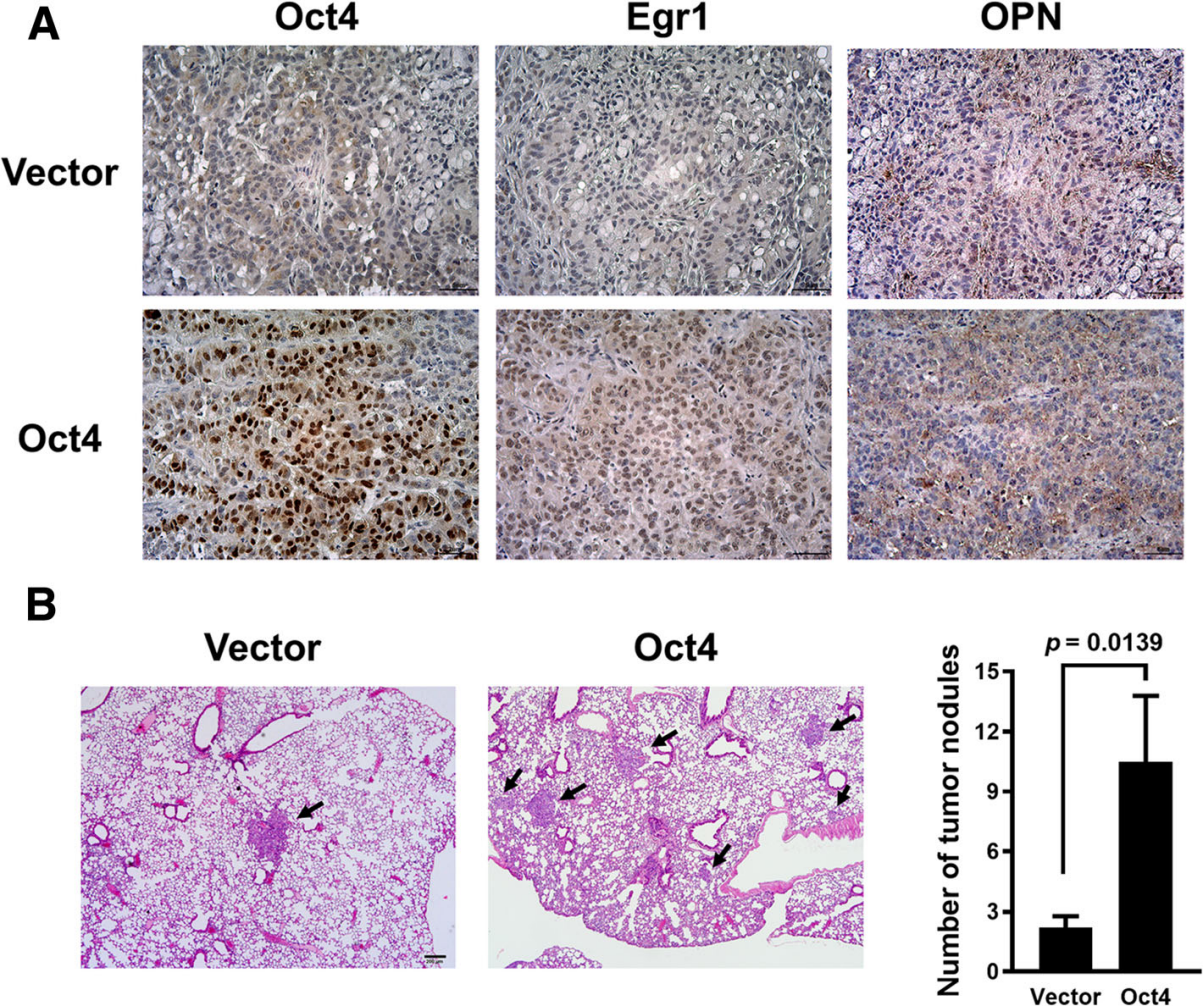
Oct4-overexpressing lung tumors express higher levels of Egr1 in a mouse tumor model, and knockdown of Oct4 reduces the migratory capability of lung cancer cells. **a**, Immunohistochemical staining for Oct4, Egr1 and OPN in the tumors excised at day 60 from NOD/SCID mice that had been inoculated with 5 × 10^6^ of A549-Oct4 cells via the tail vein. **b**, Lung tissues of tumor-bearing mice were harvested, and the number of metastatic nodules was counted. Data are mean ± SEM. *, *p* < 0.05; **, *p* < 0.01

Given that Oct4 positively regulates Egr1 expression, we next determined whether the in vitro results could be found in vivo. To this end, we injected A549-Oct4 cells subcutaneously into NOD/SCID mice and examined the expression of Oct4, Egr1 and OPN in tumor tissues by immunohistochemical staining after 60 days. Figure 4a shows that Oct4-overexpressing tumors expressed higher levels of Egr1 and OPN compared to Oct4 low-expressing tumors. Furthermore, numbers of lung tumor nodules observed by H&E staining were significantly higher in mice bearing A549-Oct4 tumors than in their control counterparts, suggesting that overexpression of Oct4 in lung cancer cells increases the metastatic potential in vivo (Fig. 4b).

**Fig. 5:**
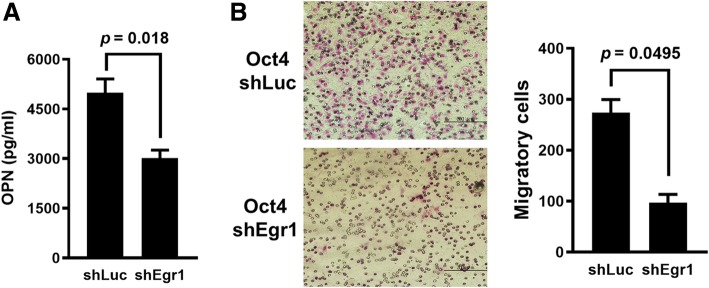
Knockdown of Egr1 reduced the levels of OPN secreted into the culture medium and decreased the migratory capability. **a**, Levels of OPN in the supernatants of Egr1 knockdown and control A549-Oct4 cells were quantified by ELISA. **b**, Migratory capabilities of Egr1 knockdown or control A549-Oct4 cells via lentivirus-mediated delivery of shRNA for 48 h. Cells that migrated through the membrane to the lower surface in the Boyden chamber were stained with Giemsa solution and visualized by light microscopy and photographed (left panel), and three fields in each membrane were counted (right panel). Data are mean ± SEM. *, *p* < 0.05; **, *p* < 0.01

We next investigated whether Egr1 was involved in the enhancing effect of Oct4 on the metastatic potential of lung cancer cells in vitro. A549-Oct4 cells were transduced with lentiviral vectors expressing Egr1 shRNA (LV.shEgr1) or with the control vector (LV.shLuc) for 3 days. Our results show that knockdown of Egr1 reduced the levels of OPN secreted into the culture medium (Fig. 5a) and decreased the migratory capability (Fig. 5b) of A549-Oct4 cells. Collectively, these results demonstrate that Oct4 can enhance the migration of lung cancer cells through Egr1.

To further validate that Oct4 controls cancer metastasis through upregulation of Egr1 in vivo, we performed lentivirus-mediated knockdown of Egr1 in A549-Oct4 and A549-Vector cells. We examined lung tissues excised from NOD/SCID mice that had been intravenously inoculated with A549-Oct4-shEgr1, A549-Oct4-shLuc, A549-Oct4, or A549-Vector cells. Our results show that overexpression of Oct4 increased metastatic lung nodules, whereas knockdown of Egr1 dramatically reduced metastatic lung nodules in an experimental metastatic cancer model (Additional file 2: Figure S1). The contribution of Oct4 in the upregulation of Egr1 and its downstream gene OPN was further verified in H1299 cells transduced with LV.shOct4 or LV.shLuc. Although the efficiency of Oct4 knockdown was only 30%, the mRNA levels of Egr1 and OPN in the Oct4 knockdown cells were 10 and 30% relative to controls, respectively (Additional file 3: Figure S2). Collectively, these results indicate that Oct4 enhances the expression of Egr1 and its downstream gene OPN, thereby contributing to cancer metastasis. 

## Discussion

The role of Egr1 in cancer cells is complex, which is dependent on the cell types and various stimuli. In colon cancer cells, overexpression of cyclooxygenase 2 significantly increases the mRNA and protein levels of microsomal prostaglandin E2 synthase 1, leading to cancer progression. Silencing of Egr1 blocks the microsomal prostaglandin E2 synthase 1 expression induced by cyclooxygeneas 2 [33]. Breast cancer cells that have undergone hypoxic conditions can induce Egr1 and hypoxia-inducible factor-1α. This can lead to breast cancer metastasis through the tissue factor pathway [34]. However, previous reports have not consistently supported the oncogenic role of Egr1. TAp73 reiterates the main part of p53 and has five distinct Egr1 binding sites. Non-consensus p53-binding sites in p73, p53, and Egr1 exhibit inter-regulating processes and sustained expression through a feedback loop, leading to prolonged expression of the *p53* gene family and efficient apoptosis [19]. Among lung cancer cell lines, H1299 cells exhibit partial homozygous deletion of the *TP53* gene, resulting in a lack of p53 protein expression and indefinite replication [35]. In contrast, A549 cells have no mutations within the *TP53* gene, but rather have a deletion on the cyclin-dependent kinase inhibitor 2A (CDKN2A) locus that harbors the *p16* and *p14ARF* genes. Mouse double minutes 2 homolog (MDM2) keeps p53 low in the absence of p14ARF [36]. Recently, a few studies have elucidated that Egr1 may be involved in cancer progression or act against cell apoptosis. In p53-deficient osteogenic sarcoma cells, the pro-survival pathway of MAPK/Akt/Nrf2/Egr1/hemoxygenase-1/glutamate cysteine ligase was shown to reduce 15-deoxy-D12,14-PGJ2 (a stable prostaglandin D2 degradation product) -mediated apoptosis [37]. Focusing on epigenetic regulation of B cell oncogenesis, combining a miR-146a deficiency with transgenic c-Myc was shown to lead to the development of highly aggressive B cell malignancies. Overexpression of mir-146a can result in down-regulation of Egr1 and downstream targets with a concomitant decrease in cell growth [38].

In the present study, we demonstrate that the expression of Oct4 is well correlated with Egr1 expression independent of the p53 status between H1299 and A549 cells. Egr1 has not been well studied in terms of the pathways involving Oct4. In the reprogramming process of pluripotent stem cells, inhibition of let-7 was found to suppress the Egr1 translation by enhancing the LIN-41 effect [39]. Other studies focusing on liver regeneration have shown that the NF-κB inhibitory protein A20 regulates the transcriptional activities of Egr1 and Oct4 [40]. There is no evidence disclosing or historically showing that Oct4 directly interacts with Egr1. In the present study, we first demonstrated the binding of Oct4 to the Egr1 promoter at − 360 bp that encompasses the Oct4 binding site. This evidence was enforced by the coexpression of Oct4 and Egr1 in the A549 mouse model. Egr1 is also involved in the regulation of homeostasis of hematopoietic cells. Mice lacking Egr1 exhibit significant increases in steady-state levels of dividing hematopoietic stem cells in their bone marrow and a striking spontaneous mobilization of hematopoietic stem cells into the peripheral blood [41]. Oct4 has been indicated to play an oncogenic role in a variety of cancers. In the present study, we further explored the biological effect of Egr1 in lung cancer cells. There is controversy in several previous reports regarding the growth inhibition of lung cancer cells by overexpression of Egr1 [42]. Our results have confirmed that in the axis of Oct4-overexpressing cancer cells, Egr1 is upregulated and has oncogenic effects on the invasiveness and metastasis of lung cancer cells both in vitro and in vivo.

Previous studies have identified a positive feedback loop in rat vascular smooth muscle cells, in which Egr1 binds to the OPN promoter and upregulates OPN expression. In addition, OPN also upregulates Egr1 expression via the ERK signaling pathway [25]. OPN, a member of the small integrin-binding ligand N-linked glycoprotein family, is expressed in normal epithelial cells of metabolically active ducts and in numerous cancers [43]. To clarify the association between OPN and stem cells, OPN has also been demonstrated to positively regulate key stemness transcription factors, including SOX2, Oct4, and Nanog [44]. Here, we demonstrate that Oct4 is involved in OPN expression through upregulating Egr1 expression in lung cancer metastasis. Egr1 is positively correlated with Oct4 and OPN in human lung cancer. All of the three biomarkers clinically favor the inferior survival nature of lung cancer.

## Conclusions

We show that the Egr1/OPN axis is regulated in Oct4-expressing lung cancer. Therapeutic strategies targeting the Oct4/Egr1/OPN axis pose a potential option for improving the grave course of lung cancer and the high resistance to systemic lung cancer treatments.

## Additional files

Additional file 1: **Table S1.** The clinico-pathological parameters of 79 lung cancer patients. (DOCX 17 kb)

Additional file 2: **Figure S1.** Knockdown of Egr-1 in Oct4-overexpressing A549 tumors reduces metastatic lung nodules in an experimental metastatic cancer model. A549 cells stably overexpressing Oct4 (A549-Oct4) and vector control cells (A549-Vector) were transduced with LV.shEgr1 and LV.shLuc to generate A549-Oct4-shEgr1 and A549-Oct4-shLuc cells. Subsequently, NOD/SCID mice were injected with 5 × 10^6^ of A549-Oct4, A549-Vector, A549-Oct4-shEgr1, or A549-Oct4-shLuc cells via the tail vein. The lungs were excised at day 60 and paraffin-embedded lung tissue sections were stained with H&E. Their macroscopic (A) and histologic (B) appearances reveal the presence of tumor nodules (indicated with arrows). (TIFF 3203 kb)

Additional file 3: **Figure S2.** Knockdown of Oct4 in lung cancer cells reduces the expression of Egr1 and OPN. H1299 cells were transduced with LV.shOct4 or LV.shLuc and their mRNA levels of Oct4, Egr1, and OPN were analyzed by real-time quantitative RT-PCR. GAPDH served as the quantitative control. Expression levels of the control cells (H1299-shLuc) were set to 100. (TIFF 403 kb)

## Data Availability

The datasets generated during and/or analyzed during the current study are available from the corresponding author on reasonable request.

## Abbreviations

Egr1: Early growth response gene 1
OPN: Osteopontin
ORE: Oct4 response element
