# Supplementary material for: Oct4 upregulates osteopontin via Egr1 and is associated with poor outcome in human lung cancer
Source: BMC Cancer. 2019 Aug 9;19:791. doi: 10.1186/s12885-019-6014-5 (PMC6688208; doi:10.1186/s12885-019-6014-5)
Supplement: Supplementary file 1 — Table S1. The clinico-pathological parameters of 79 lung cancer patients. (DOCX 17 kb) [file 12885_2019_6014_MOESM1_ESM.docx]

Table S1. The clinico-pathological parameters of 79 lung cancer patients.

| Case number | 79 |
| --- | --- |
| Age, year  median (range) | 73 (49 – 90) |
| Sex  male  female | 46  33 |
| Tumor pathological stage  I  II  III  IV | 38  20  20  1 |
| Lymph node involvement  negative  positive | 41  38 |
| Operative method  wedge resection  lobectomy  pneumonectomy | 2  70  7 |
| Oct4 expression  negative  weak  moderate  strong | 4  33  21  21 |
| Egr1 expression  negative  weak  moderate  strong | 1  38  25  15 |
| OPN expression  negative  weak  moderate  strong | 1  33  26  19 |
