# Supplementary figures and images for: Oct4 upregulates osteopontin via Egr1 and is associated with poor outcome in human lung cancer
Source: BMC Cancer. 2019 Aug 9;19:791. doi: 10.1186/s12885-019-6014-5 (PMC6688208; doi:10.1186/s12885-019-6014-5)

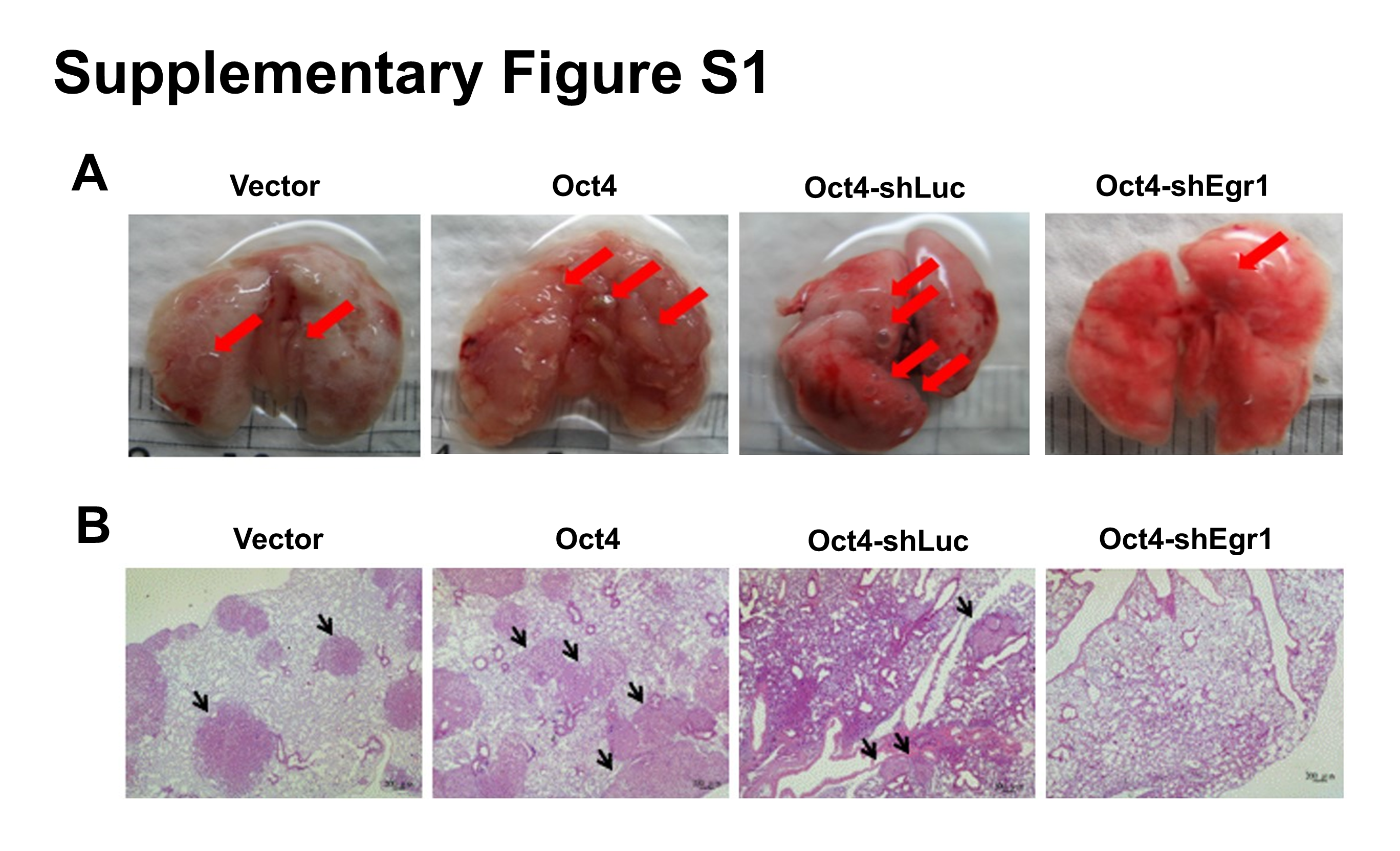

Supplement: Supplementary file 2 — Figure S1. Knockdown of Egr-1 in Oct4-overexpressing A549 tumors reduces metastatic lung nodules in an experimental metastatic cancer model. A549 cells stably overexpressing Oct4 (A549-Oct4) and vector control cells (A549-Vector) were transduced with LV.shEgr1 and LV.shLuc to generate A549-Oct4-shEgr1 and A549-Oct4-shLuc cells. Subsequently, NOD/SCID mice were injected with 5 × 10^6^ of A549-Oct4, A549-Vector, A549-Oct4-shEgr1, or A549-Oct4-shLuc cells via the tail vein. The lungs were excised at day 60 and paraffin-embedded lung tissue sections were stained with H&E. Their macroscopic (A) and histologic (B) appearances reveal the presence of tumor nodules (indicated with arrows). (TIFF 3203 kb) [file 12885_2019_6014_MOESM2_ESM.tiff]

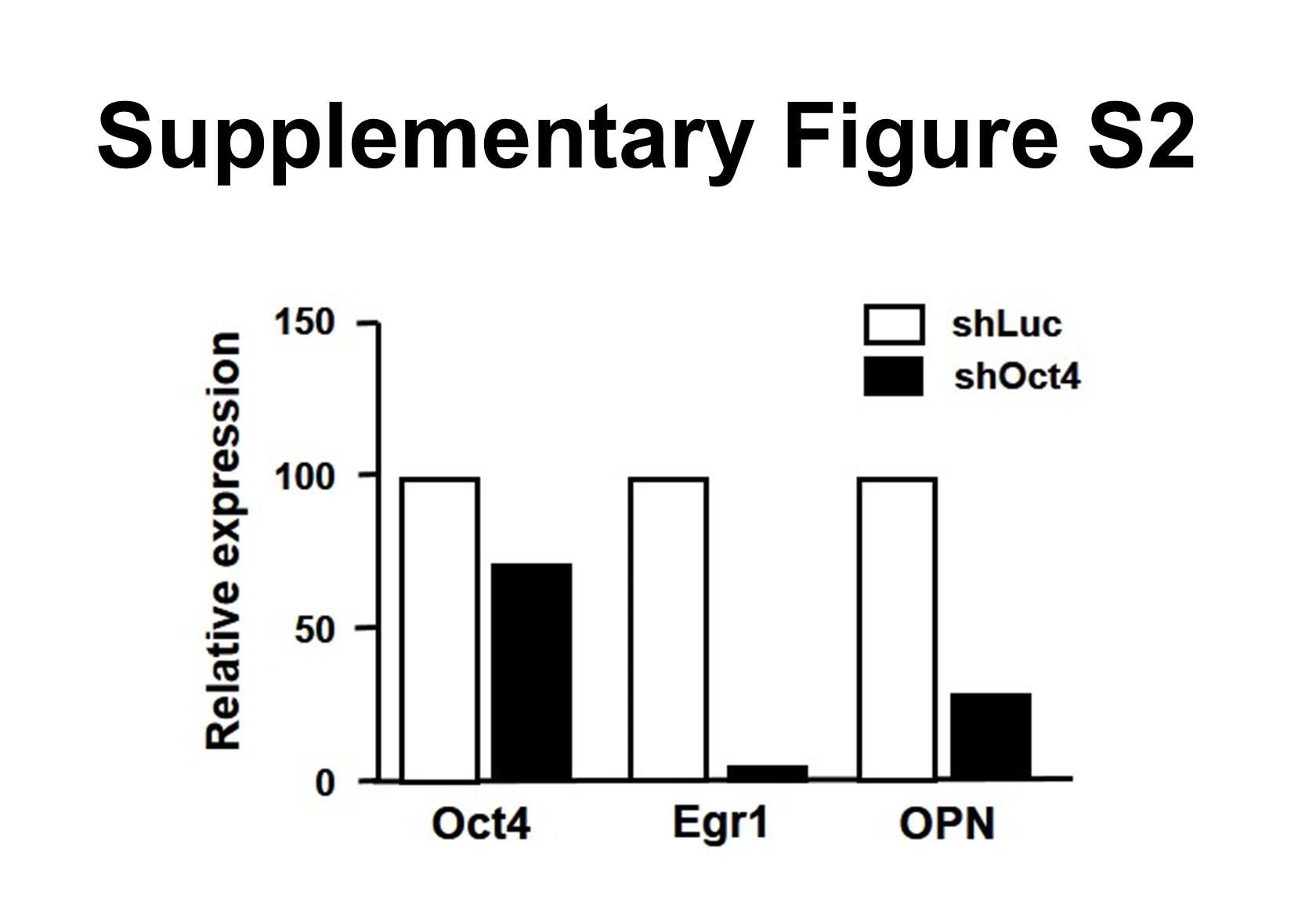

Supplement: Supplementary file 3 — Figure S2. Knockdown of Oct4 in lung cancer cells reduces the expression of Egr1 and OPN. H1299 cells were transduced with LV.shOct4 or LV.shLuc and their mRNA levels of Oct4, Egr1, and OPN were analyzed by real-time quantitative RT-PCR. GAPDH served as the quantitative control. Expression levels of the control cells (H1299-shLuc) were set to 100. (TIFF 403 kb) [file 12885_2019_6014_MOESM3_ESM.tiff]
